# Subtle changes in topsoil microbial communities of drained forested peatlands after prolonged drought

**DOI:** 10.1111/1758-2229.70041

**Published:** 2024-11-07

**Authors:** Oona Hillgén, Marjo Palviainen, Annamari Laurén, Mari Könönen, Anne Ojala, Jukka Pumpanen, Elina Peltomaa

**Affiliations:** ^1^ Department of Forest Sciences University of Helsinki Helsinki Finland; ^2^ School of Forest Sciences, Faculty of Science, Forestry and Technology University of Eastern Finland Joensuu Finland; ^3^ Natural Resources Institute Finland Joensuu Finland; ^4^ Department of Environmental and Biological Sciences University of Eastern Finland Kuopio Finland

## Abstract

A major consequence of anthropogenic climate change is the intensification and extension of drought periods. Prolonged drought can alter conditions in drained peatlands and cause disturbances in microbial communities in the topsoil layer of the peat. Varying environmental conditions throughout the growing season, such as the availability of organic matter and nutrients, temperature and water table, further impact these communities and consequently affect carbon and nutrient cycles. The impact of drought and new forestry practices is largely unknown in drained peatland forests. We examined how microbial communities change over a growing season in different harvesting intensities (continuous cover forestry, clear‐cut and uncut) in a drained peatland site using bacterial 16S and fungal ITS2 rRNA analysis. We found seasonal differences in bacterial and fungal diversity and species richness, and subtle changes in microbial communities at the phylum and genus levels when comparing various environmental factors. Diversity, species richness and relative abundance differed in spring compared to summer and autumn. However, significant differences in the microbial community structure were not detected. Understanding the responses of microbial communities to disturbances like drought and other environmental factors provides new insights into the consequences of climate change on drained forested peatlands.

## Graphical abstract

This study examined how microbial communities in drained peatlands respond to seasonal changes and drought under different harvest intensities (continuous cover forestry, clear‐cut and uncut). Using bacterial 16S and fungal ITS2 rRNA analysis, we observed shifts in diversity and species richness between seasons. No major changes in microbial community structure were found. These findings provide insight into how climate change and drought may affect microbial communities and their functions in drained forested peatlands.

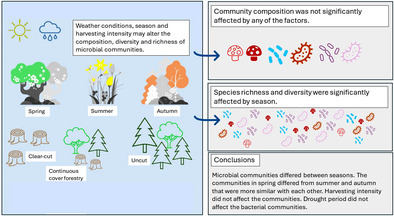

## INTRODUCTION

Climate change is predicted to increase the risk of summer droughts and other extreme weather conditions. These changes may alter the composition, structure and function of soil microbial community and enzyme production, and ultimately affect soil C and nutrient cycling (Bogati & Walczak, 2022; Li et al., 2024; Venäläinen et al., 2020; Xu et al., 2021). The impact of drought varies with the soil type, vegetation, depth, season and microbe characteristics (Cordero et al., 2023; de Souza et al., 2024; Lamit et al., 2021; Peltoniemi et al., 2009; Veach et al., 2020; Wang, Meister, et al., 2022; Xu et al., 2021; Yang et al., 2024). The severity of drought is affected by evaporation, transpiration and interception, which in turn depend on the forest stand characteristics such as stand volume and leaf mass (Launiainen et al., 2016). In peatlands, different layers of peat and surface vegetation can impact the microbial responses to drought significantly. The shift in communities caused by these factors is more visible in the top layers of the peat than in the deep layers (Lamit et al., 2021). Lowering of the water table (WT) has been shown to benefit fungi and increase bacterial and fungal biomass (Andersen et al., 2013; Jaatinen et al., 2008), resulting in more efficient aerobic decomposition. Bacteria and fungi are responsible for organic matter decomposition, but fungi have stronger links to plants through mycorrhiza, whereas the relationships between plants and bacteria are less tight (Baldrian, 2017; Mundra et al., 2022). In controlled laboratory conditions, drought has caused changes in the community composition of microbes in peat (Potter et al., 2017). The study by Peltoniemi et al. (2009) included drained peatland sites, where drought was noticed to have changes in communities, especially in fungi and Actinobacteria. However, the overall effects of drought on microbial communities are poorly understood in boreal‐drained forested peatlands.

Boreal forests and peatlands are globally significant reservoirs of carbon (C) (Bradshaw & Warkentin, 2015; Wieder et al., 2006). This function is affected by weather conditions, forest management and climate change (Charman et al., 2013; Harenda et al., 2018). Weather conditions and forest harvesting induce changes in soil temperature and moisture, WT, oxygen availability, soil pH and the amount and quality of organic matter input (Baldrian, 2017; Briones et al., 2014; Jaatinen et al., 2008; Keiluweit et al., 2016; Laiho, 2006; Peltoniemi et al., 2009; Peltomaa et al., 2022). In Finland, nearly half of the original peatland area (10 M ha) has been drained for forestry, majority of which was drained for the first time 50–60 years ago. Drainage improves tree growth, increases peat decomposition, alters the amount and species composition of ground vegetation and onsets the formation of raw humus layer over the original peat (Kaunisto & Moilanen, 1998; Laiho, 1997). The raw humus layer contains a significant nutrient pool (Kaunisto & Moilanen, 1998) and the majority of the fine roots of trees are concentrated in this layer (Lampela et al., 2023; Wei et al., 2023). The physical and chemical characteristics of the raw humus layer are similar to those of mor, an organic soil layer found in upland mineral soils (Laurén, 1999). Microbial communities in pristine peat and upland mor layers have been studied (Andersen et al., 2013; Kitson & Bell, 2020), but studies on microbial communities in raw humus of drained peatlands are scarce. The large amount of horizontally oriented macropores in the humus layer restricts the capillary rise of water from the underlying WT to the humus layer. This maintains good aeration in the root layer during wet periods. However, it also makes the humus layer vulnerable to drought during dry periods, which can disrupt microbe‐mediated nutrient cycling. Therefore, it is essential to study the drought‐induced changes in microbial communities, as the supply of nutrients from decomposing organic material is the most important factor regulating forest growth in drained peatlands (Laurén et al., 2021).

Peatland forest management, such as drainage and harvesting, induces differences in the vegetation, which in turn affects the microbial communities due to changes in organic matter input (Laiho, 2006; Peltoniemi et al., 2009). Forest stand characteristics, including tree species, leaf mass and basal area, can affect the interception capacity (Launiainen et al., 2016) and evapotranspiration and therefore also affect WT and drought intensity. Mature spruce stands with high leaf mass are particularly vulnerable to drought (Netherer et al., 2024). Activities like harvesting and drainage can affect the WT, oxygen availability, organic matter quality and chemical properties of soil (Peltomaa et al., 2022). Furthermore, new forest management methods such as continuous cover forestry (CCF) are becoming more common. The CCF preserves part of stand and ground vegetation, maintaining more stable soil moisture and temperature conditions and maintains a higher potential for C accumulation than clear‐cut or uncut forests. Therefore, CCF can be expected to cause fewer changes in the microbial community than clear‐cutting (Kim et al., 2021; Roth et al., 2023).

The rRNA transcripts have been used to identify active populations in mixed microbial communities (Blazewicz et al., 2013; Salgar‐Chaparro & Machuca, 2019). By adapting these methods, we can obtain a snapshot of the microbial functional groups in the soil. However, using rRNA transcripts to determine the microbial community does not fully capture the active community, as rRNA represents the potential for the activity, rather than the activity itself (Blazewicz et al., 2013). Furthermore, when studying only the active community, the samples often contain 16S rRNA genes from dead or dormant cells (Li et al., 2017). As a part of the ribosome, rRNA is involved in cell physiology and changes, and therefore can be linked to the community members that are or have recently been active (Gourse et al., 1996; Kerkhof & Ward, 1993; Poulsen et al., 1993).

This study aimed to examine the effects of drought, seasonal changes and forest harvesting intensity on bacterial and fungal communities in the topsoil layer consisting of raw humus and surface peat of dried peatland forest over a growing season. During the experiment, there was a prolonged drought period in the summer. Our Norway spruce‐dominated study sites were treated with clear‐cut, selective cutting (CCF), whereas one of them was left uncut providing us with a set of different moisture regimes within the same study area. We used bacterial 16S and fungal ITS2 rRNA analysis to discover the present active community members. We hypothesized that (1) the microbial communities vary seasonally and respond to drought, (2) the bacterial and fungal communities show different responses to drought and (3) their responses are dependent on forest stand characteristics.

## EXPERIMENTAL PROCEDURES

### Site description and sampling

Soil samples were collected from a nutrient‐rich drained peatland forest dominated by Norway spruce (*Picea abies* (L.) Karst.) in Paroninkorpi (61.01° N, 24.75° E) in Southern Finland. The ditch network was established at the beginning of the 1960s and underwent ditch cleaning in 2018. The current ditch spacing is 50 m, the depth is ca. 0.6 m, and the peat deposit is >1.5 m deep and formed of a thin raw humus layer (5–10 cm) overlaying moderately decomposed *Carex*‐wood peat. The site was divided into plots (40 × 40 m) representing three harvesting intensities: (1) clear‐cut (all trees removed, basal area 0 m^2^ ha^−1^), (2) CCF (basal area 12 m^2^ ha^−1^) and (3) uncut forest (the basal area 25 m^2^ ha^−1^). The harvesting was conducted when the thick snow cover protected the ground vegetation and soil in February 2017. The ground vegetation was formed by dwarf shrubs (*Vaccinium myrtillus* L., *V. vitis‐idaea* L.), mosses (mainly *Pleurozium schreberi* Brid., *Hylocomium splendens* (Hedw.). Schimp. and some *Sphagnum* sp.), and ferns in the CCF and uncut plots. In the clear‐cut plots, thick patches of raspberry (*Rubus idaeus* L.) and birch (*Betula* sp.) had taken over the site, along with young Norway spruce seedlings planted in 2018. The pH, C and N content and C:N ratio of the peat in management plots are shown in Table 1 to provide background information on the area's characteristics. More detailed descriptions of the study site can be found in Palviainen et al. (2022) and Peltomaa et al. (2022).

**TABLE 1:** The pH, carbon (C) and nitrogen (N) content as well as the C:N ratio of the peat in 2021 in the clear‐cut, continuous cover forestry (CCF) and uncut forest plots (data from the Natural Resources Institute Finland).

Forest management	pH	C (g kg^−1^)	N (g kg^−1^)	C:N‐ratio
Clear‐cut	4.04 (0.18)	51 (4.8)	1.9 (0.2)	27 (2.7)
CCF	3.90 (0.19)	51 (4.4)	2.2 (0.2)	23 (2.7)
Uncut	3.76 (0.16)	47 (2.5)	1.9 (0.2)	24 (2.5)

*Note*: Standard deviations are given in parenthesis.

Soil samples for the soil microbial community analysis were collected from the surface soil including raw humus and surface peat (0–10 cm) in the spring (May), summer (July), and autumn (September) of 2021. The samples with three replicates were taken 1 m apart from each other in the middle of each plot, ca. 20 m from the ditches. The sampling placement (80 m between the plots) was selected to avoid edge effects from different directions (at least 2 × height of the trees) and for standardization of drainage effect (distance from the ditches). The ground vegetation was removed prior to sampling. Soil cores were collected using a cylinder sampler (diameter 3 cm). The soil was homogenized in a sterilized container by shaking, and then transferred to a sterilized 50 mL plastic tube, preserved with DNA/RNA Shield (Zymo Research, CA, USA), and placed on ice. The tubes were stored at −20°C before RNA extraction in the laboratory. Soil temperature in the field was measured at a depth of 10 cm using a digital stick thermometer (Orthex Group, Finland). The WT was monitored from groundwater tubes installed down to 1 m depth (see Palviainen et al., 2022 for further details) located within a 1–2 m distance from the soil sampling points. Weather data was collected ca. 20 km from the study site (Lammi Pappila weather station), by the Finnish Meteorological Institute.

### RNA extraction and sequencing

RNA was extracted from the soil samples using the RNA PowerSoil® Total RNA Isolation Kit (Qiagen, Ireland) following the manufacturer's instructions. RNA concentration was verified using the Qubit RNA High Sensitivity RNA Assay Kit (Invitrogen, Life Technologies, CA, USA) and Qubit 2.0 fluorometer (Invitrogen). Complementary DNA (cDNA) was synthesized using the Quantinova Reverse Transcription Kit (Qiagen).

The samples were sequenced by Novogene Company Ltd. (UK) using a sequencing depth of 100 K raw tags (a recommendation for complex data such as soil samples). For the amplicon generation, the bacterial V3‐V4 region of the 16S rRNA gene was amplified using the primer pair 341F/806R (Table 2; Herlemann et al., 2011), whereas the primer pair ITS3‐2024F/ITS4‐2409R (Table 2; Bellemain et al., 2010) was used for the fungal internal transcribed spacer 2 (ITS2) region. The PCR products were selected by 2% agarose gel electrophoresis, end‐repaired, A‐tailed and further ligated with Illumina adapters. The libraries were sequenced on a paired‐end Illumina platform to generate 250‐bp paired‐end raw reads.

**TABLE 2:** Primers used in amplicon sequencing of the bacterial 16S and the fungal internal transcribed spacer 2 (ITS2) regions.

Amplified region	Fragment length	Primers	Sequences (5′‐3′)
Bacterial 16S V3‐V4	470 bp	341F	CCTAYGGGRBGCASCAG
		806R	GGACTACNNGGGTATCTAAT
Fungal ITS2	380 bp	ITS3‐2024F	GCATCGATGAAGAACGCAGC
		ITS4‐2409R	TCCTCCGCTTATTGATATGC

Samples were further processed by using QIIME2 (Version 2024.5; Bolyen et al., 2019). The primer sequences (Table 2) were discarded by using Cutadapt (Martin, 2011). DADA2 (Callahan et al., 2016) pipeline was used to detect and correct Illumina amplicon sequence data, filter any phiX reads and chimeric sequences and merge paired reads. It should be noted that whereas bacterial sequences were run with forward and reverse sequences, fungal sequences were only run with forward sequences. This was done due to the low quality of the autumn samples. The spring samples were good quality. The bacterial sequences were truncated at 170 bp (forward) and 200 (reverse). Fungal sequences were truncated at 220 bp. The truncation was decided based on the quality of the samples. The tree for phylogenetic diversity analyses was created by using QIIME2 phylogeny plugin. Taxonomic analysis was done using pre‐trained classifiers. For bacteria, SILVA 138 SSU database and classifier were used (Robeson et al., 2020; https://www.arb-silva.de) and for fungi UNITE v.10.0 (Version 04.04.2024; Abarenkov et al., 2024; https://unite.ut.ee/). The classification was done using scikit‐learn (Pedregosa et al., 2011) and the sequences were assigned to ASVs (amplicon sequencing variants).

### Statistical analyses

All statistical testing was performed with R software (version 4.3.2). Statistical testing, data normalization, rarefaction, diversity and richness analyses and examination of community composition were performed using Phyloseq (version 1.46.0). In statistical testing, *p*‐values <0.05 were considered significant. Data was rarefied (minimum sequencing depth reduced by 10%) and alpha diversity indices, observed species, species richness (Chao1; Chao, 1984) abundance‐based coverage estimator (ACE; Chao & Lee, 1992) and diversity (Shannon index; Shannon, 1948 and Simpson index; Simpson, 1949) were calculated. The normality of the data was checked using Shapiro–Wilk test (Shapiro & Wilk, 1965) and Q‐Q plots. Differences in alpha diversity between seasons and harvest intensity were then examined using the Kruskal‐Wallis test (Kruskal & Wallis, 1952), as data was not normally distributed and included multiple groups. Differences were then visualized using a boxplot. Lastly, the differences were further examined using the Wilcoxon rank‐sum test (Mann & Whitney, 1947). Principle coordinate analysis (PCoA) plots were created using Bray–Curtis dissimilarity matrix to visualize community‐level sample dissimilarity (beta diversity). Dominant taxa were calculated for bacterial and fungal phyla. The relative abundance of bacterial and fungal phyla was visualized using barplots. The hierarchical clustering of the relative abundance of the most common 35 bacterial and fungal genera was examined using a hierarchical clustering heatmap. Statistical differences in beta diversity and relative abundances on a phylum and genus level in relation to multiple environmental factors (season, harvest intensity, WT, pH and soil temperature) were examined using non‐parametric permutational multivariate analysis of variance (PERMANOVA) ‘adonis2’ function of the vegan package (version 2.6‐6.1). Relationships between the samples were examined and visualized using the UpSet plot.

## RESULTS

### Weather and WT

The average air temperature of the preceding month before samplings was 3.69°C in spring, 20.0°C in summer and 14.0°C in autumn. The precipitation sums for the corresponding times were 27.5, 34.3, and 115.5 mm, respectively. The daily weather data is presented in Figure S1. The low summertime precipitation was reflected in the WT, which was significantly lower (*p* <0.05) during summer than in spring sampling (Table 3). Additionally, the soil temperatures were higher in summer than in spring in all plots (*p* <0.05) (Table 3). According to the Finnish Meteorological Institute, the summer of 2021 was the second warmest in statistics from the beginning of the 20th century for the whole country. From June to August, the average number of hot weather days (>25°C) for the whole country was 50, while the normal average for the same period is 33 days. The average temperature during this period in the study area was 21°C. From the last days of June to mid‐July, there was almost no rainfall at all in the study area. The average temperature during this period in the study area was 21°C. The highest daily average temperature during the same period was 24.5°C and the lowest 18°C. This period consists of 18 days with only one with 2.2 mm of precipitation and 11 consecutive days without precipitation. The average precipitation during summer was quite typical for the whole country. However, the precipitation did not spread evenly over the summer, and there were periods with almost no rainfall (June and July) and heavy rain (August).

**TABLE 3:** Soil temperatures and water table (WT) in the clear‐cut, continuous cover forestry (CCF) and uncut in spring, summer and autumn sampling.

Sampling	Forest management	Soil temperature (°C)	(WT, positive down; cm)
Spring	Clear‐cut	6	49
	CCF	4	42
	Uncut	3	58
Summer	Clear‐cut	25	99
	CCF	18	90
	Uncut	17	102
Autumn	Clear‐cut	12	69
	CCF	12	41
	Uncut	11	62

### 16S‐rRNA and IT2‐rRNA gene sequencing results

Amplicon sequencing of bacterial 16S rRNA sequences resulted in, on average, 73,467 effective and 71,386 annotated tags (Figure S2, Table S1). However, due to the low amount of bacterial RNA, the 16S sequencing could not be performed for one clear‐cut sample in the spring, one CCF sample in the summer and two uncut forest samples in the autumn. The amplicon sequencing of fungal ITS2 rRNA sequences resulted, on average, in 83,751 effective and 60,518 annotated tags (Figure S2, Table S1). However, none of the summer samples contained enough high‐quality fungal RNA for sequencing. Like the bacterial RNA, the fungal RNA was low for one clear‐cut and one CCF sample in the spring, and two clear‐cut samples in the autumn, resulting in no ITS2 sequencing. Further analysis produced 10,317 bacterial and 5660 fungal ASVs.

Good's coverage ranged from 95% to 99% (Table S2), indicating that most of the bacterial and fungal types have been detected in the samples. ASV classification resulted in 53 phyla, 137 classes, 296 orders, 477 families and 789 genera for bacteria and 12 phyla, 23 classes, 35 orders, 42 families and 39 genera for fungi.

### Bacterial and fungal community richness and diversity

**FIGURE 1:**
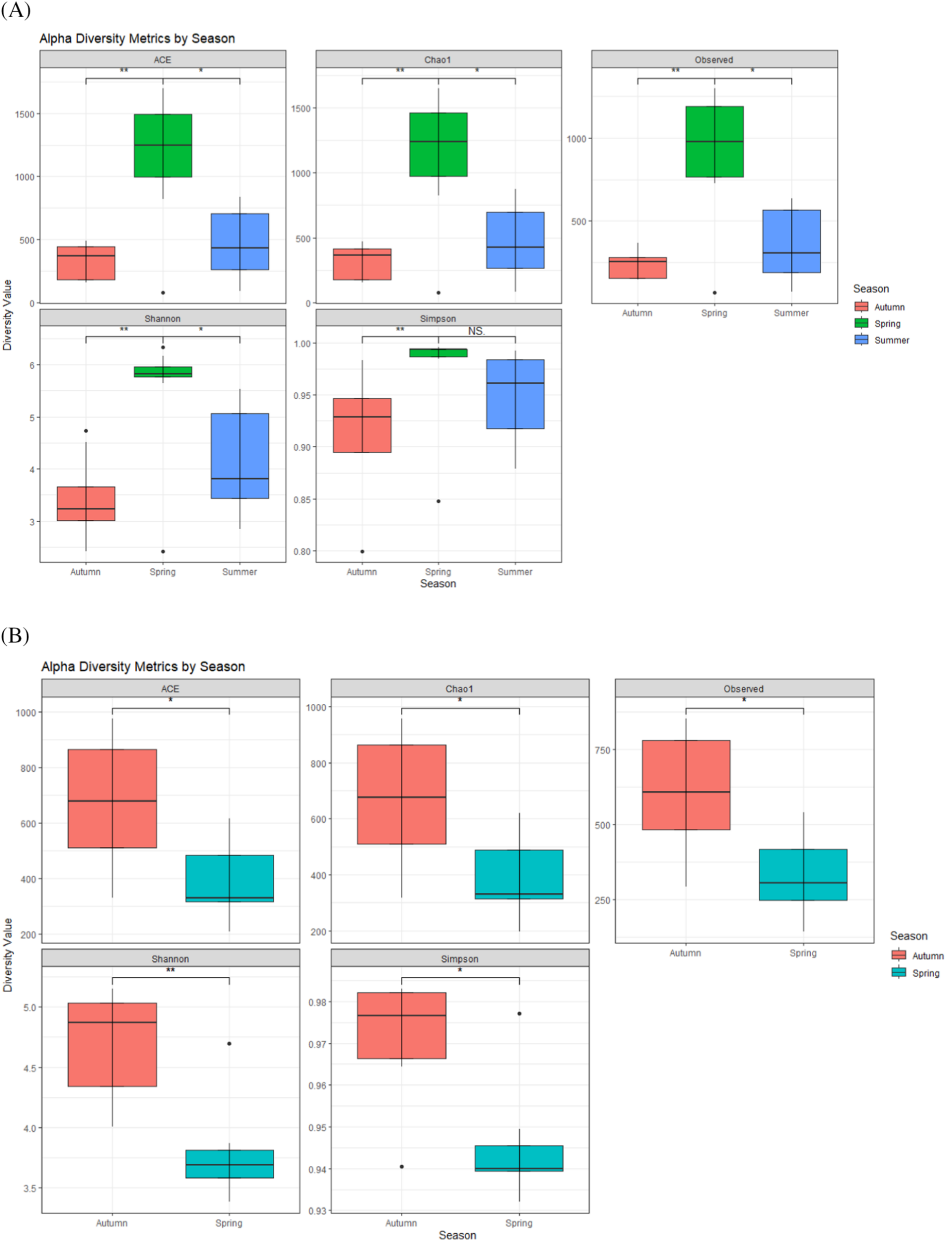
Observed species and alpha‐diversity indices. ACE and Chao1 indices represent amplicon sequencing variant (ASV) abundances in the samples. The higher index of ACE and Chao1 indicates higher expected species richness. Shannon and Simpson indices represent the diversity of ASV in the samples. A higher Shannon index indicates higher diversity, whereas a smaller Simpson index indicates higher diversity. The observed species is named ‘Observed’. The asterisks mark statistical significance (*p <0.05, **p <0.01).

Overall, species richness and diversity were highest in spring compared to summer and autumn. Examining observed species, alpha diversity (Shannon and Simpson) and species richness (Chao1 and ACE) revealed no statistical differences between bacterial or fungal richness and diversity and harvest intensities. Observed species and diversity and richness were highest in spring for bacteria and in autumn for fungi (Table S2; Figure 1). Additionally, statistical differences (*p* <0.05) could be detected between seasons. For bacteria, comparing spring to summer and autumn resulted in significant statistical differences, but there were no statistical differences between autumn and summer, and in Simpson analysis between spring and summer (Figure 1A). For fungi, the difference in species richness and diversity was also statistically significant between spring and autumn (Figure 1B).

**FIGURE 2:**
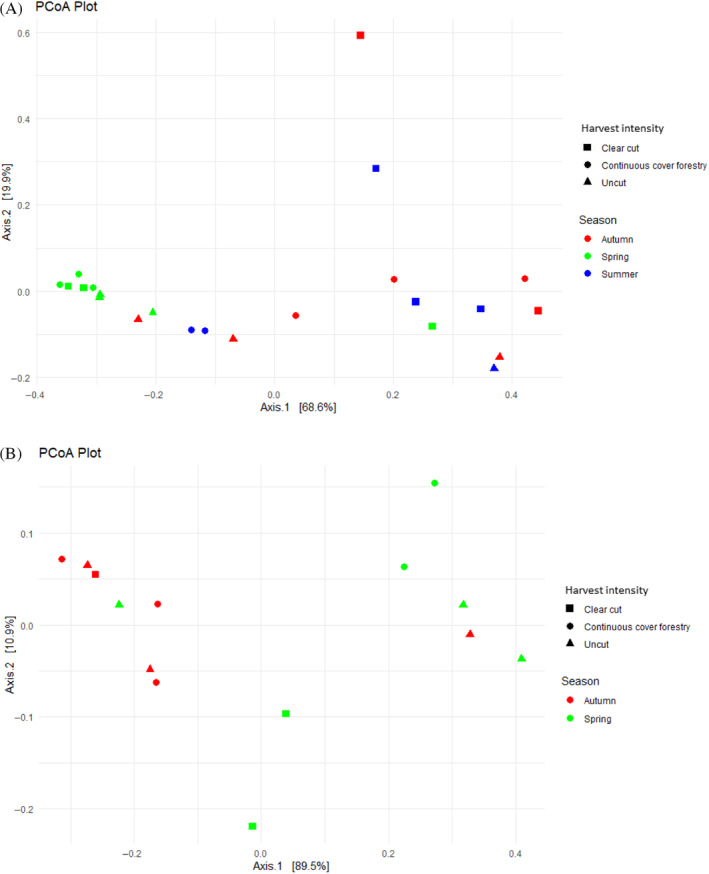
Principle coordinate analysis (PCoA) plots of the (A) bacterial and (B) fungal communities in the differently managed forests (clear‐cut, continuous cover forestry and uncut) in spring, summer and autumn. Each data point in the graph stands for a sample. The distance between the samples indicates the level of difference. The closer the samples are in the graph, the higher the similarity.

PCoA analysis (beta diversity) showed dissimilarity in microbial communities. There was a distinct structure in bacteria between spring samples and autumn and summer samples (Figure 2A). Statistical testing revealed a highly significant difference (*p* <0.01) between seasons in bacterial beta diversity. Likewise, PCoA analysis revealed a distinct structure between spring and autumn in fungal beta diversity (Figure 2B). Fungal communities also showed significant differences in beta diversity (*p* <0.05) between seasons but not as strongly as bacteria.

### Bacterial and fungal community composition

**FIGURE 3:**
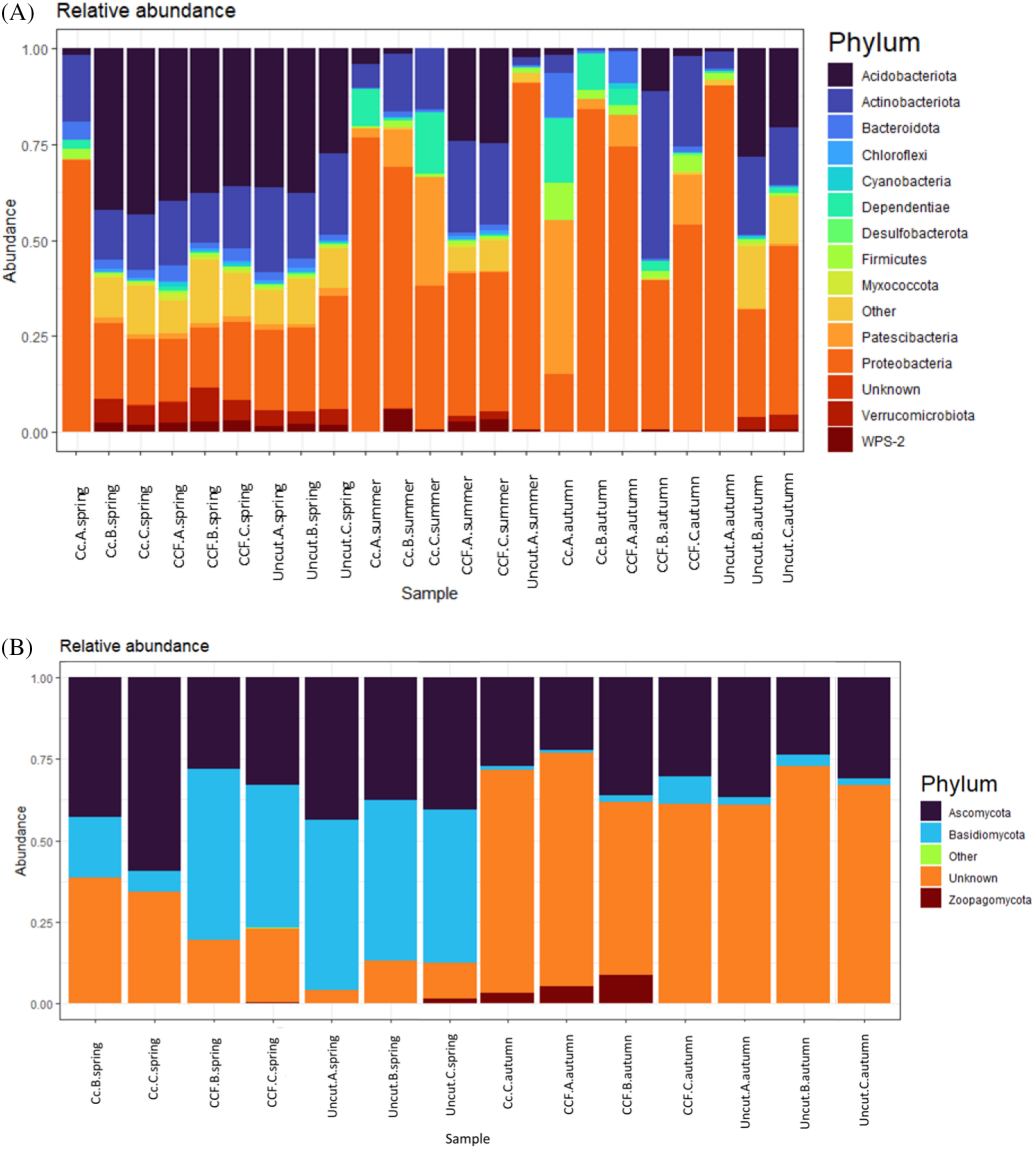
The relative abundance of (A) bacterial and (B) fungal phyla across seasons (spring, summer and autumn) and harvest intensities (clear‐cut [CC], continuous cover forestry [CCF] and uncut). A bar represents one sample. Letters A, B and C indicate sample replicates. NB, None of the summer samples contained enough high‐quality fungal RNA for sequencing and are not included.

The most common phyla for bacteria and fungi according to relative abundance in different samples are presented in Figure 3. For fungi, unassigned phyla were largely present in the samples. The most abundant bacteria in the phylum level were Acidobacteriota, Proteobacteria and Actinobacteria. The most abundant fungal phyla were Basidiomycota and Ascomycota. In spring, Acidobacteriota accounted for 77.8% of the dominant taxa, followed by Proteobacteria (22%). During summer, Proteobacteria dominated all the samples and accounted for 100% of the dominant taxa. Proteobacteria was also the most dominant in autumn (62%) but was accompanied by Acidobacteria and Actinobacteria with 12.5% share each. The fungal communities were dominated by Basidiomycota and Ascomycota. In spring, Basidiomycota covered 57.1% of the dominant taxa, whereas Ascomycota covered 28.6%. However, during autumn, Basidiomycota covered only 14.3% and Ascomycota was not among the dominant taxa. Unassigned phyla were significant in both seasons, accounting for 14.3% in spring and 85.7% in autumn. The most dominant bacterial phyla in all harvest intensities were Proteobacteria and Acidobacteriota. In clear‐cut, Proteobacteria covered 62.5% and 25% of dominant taxa, in CCF the equivalent percentages were 50% and 38%, and in the uncut site 57.1% and 42.9%. Dominant fungal communities in clear‐cut were Ascomycota (66.7%), in CCF Basidiomycota (40%) and in uncut Basidiomycota (50%). Additionally, unknown phyla were dominant in all harvest intensities (in clear‐cut 33%, in CCF 60% and in uncut 50%). Permutational multivariate analysis of variance did not reveal statistical significance between season or harvesting intensity on a phylum level. Only bacterial phylum, Myxococcota showed statistical significance when it comes to harvest intensity (*p* <0.05).

**FIGURE 4:**
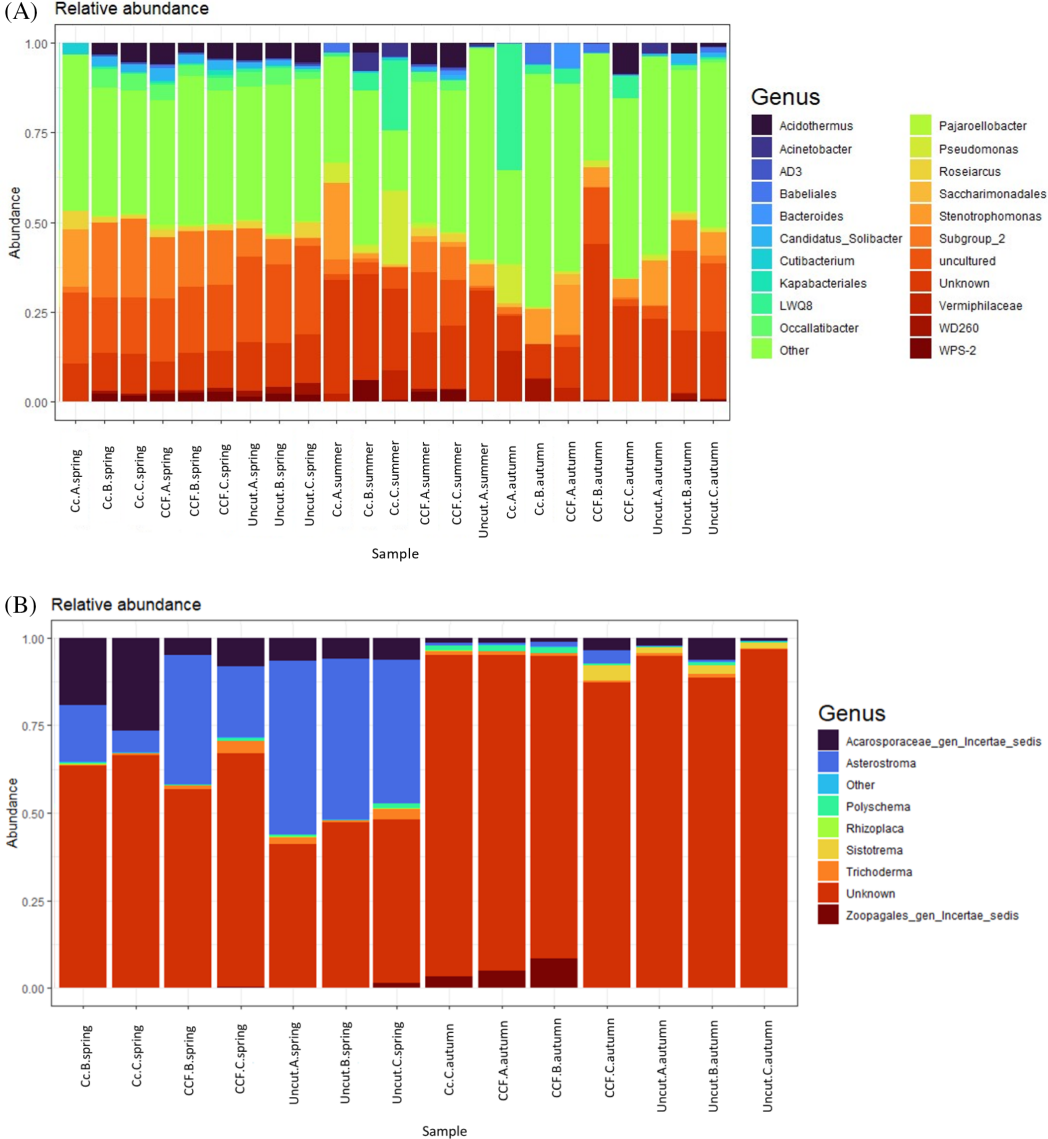
The relative abundance of (A) bacterial and (B) fungal genera across seasons (spring, summer and autumn) and harvest intensities (clear‐cut [CC], continuous cover forestry [CCF] and uncut). A bar represents one sample. Letters A, B and C indicate sample replicates. NB, None of the summer samples contained enough high‐quality fungal RNA for sequencing and are not included.

**FIGURE 5:**
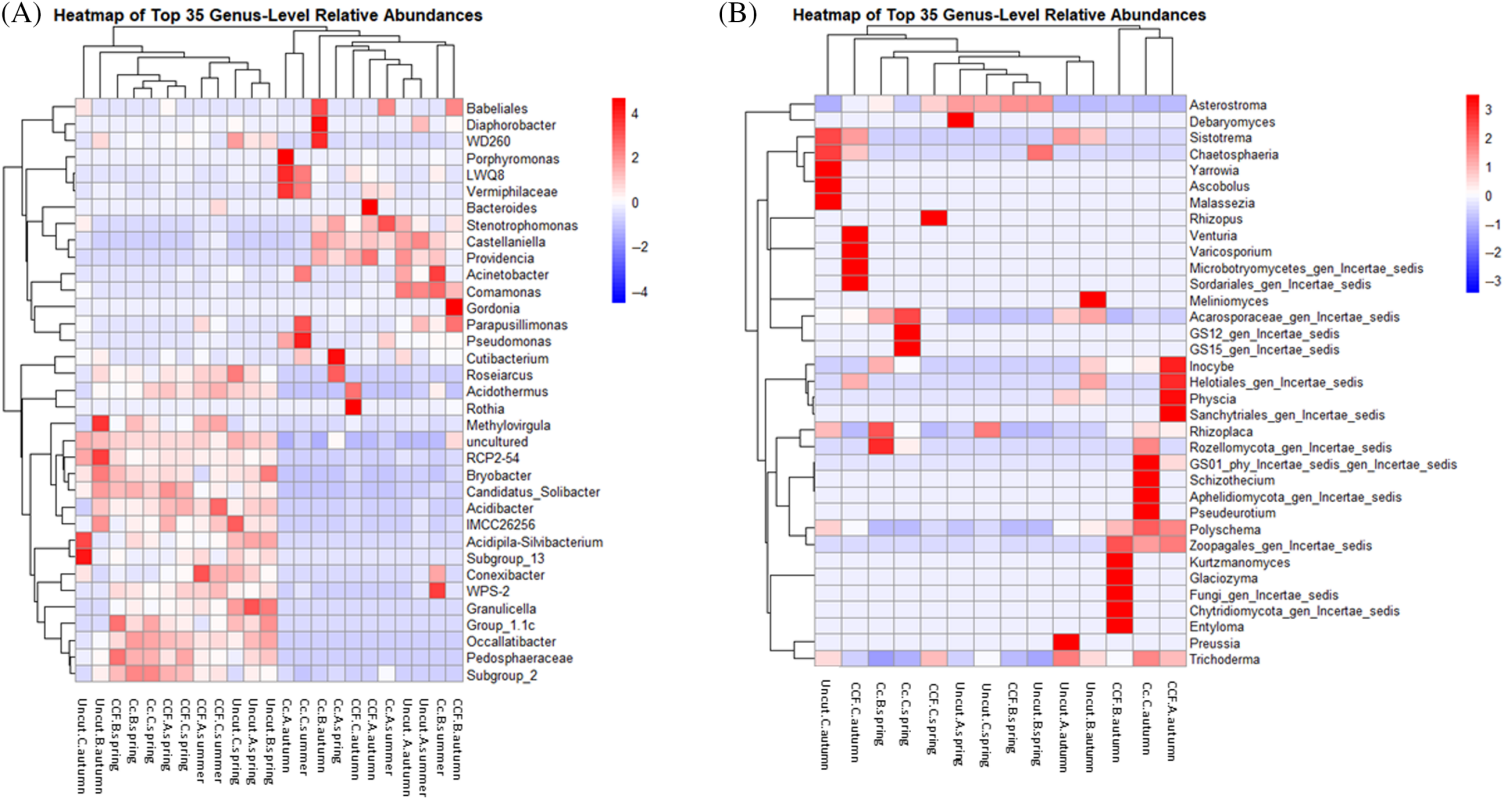
The heatmap of the hierarchical clustering of samples based on the relative abundance of the top‐ranked 35 genera of (A) bacteria and (B) fungi in the differently managed forests in the spring, summer and autumn (plotted by sample name on the X‐axis). The heatmap grid shows the abundances: Blue colours indicate lower relative abundance, while red indicates higher relative abundance. CC, clear‐cut; CCF, continuous cover forestry. Letters A, B and C indicate sample replicates.

**FIGURE 6:**
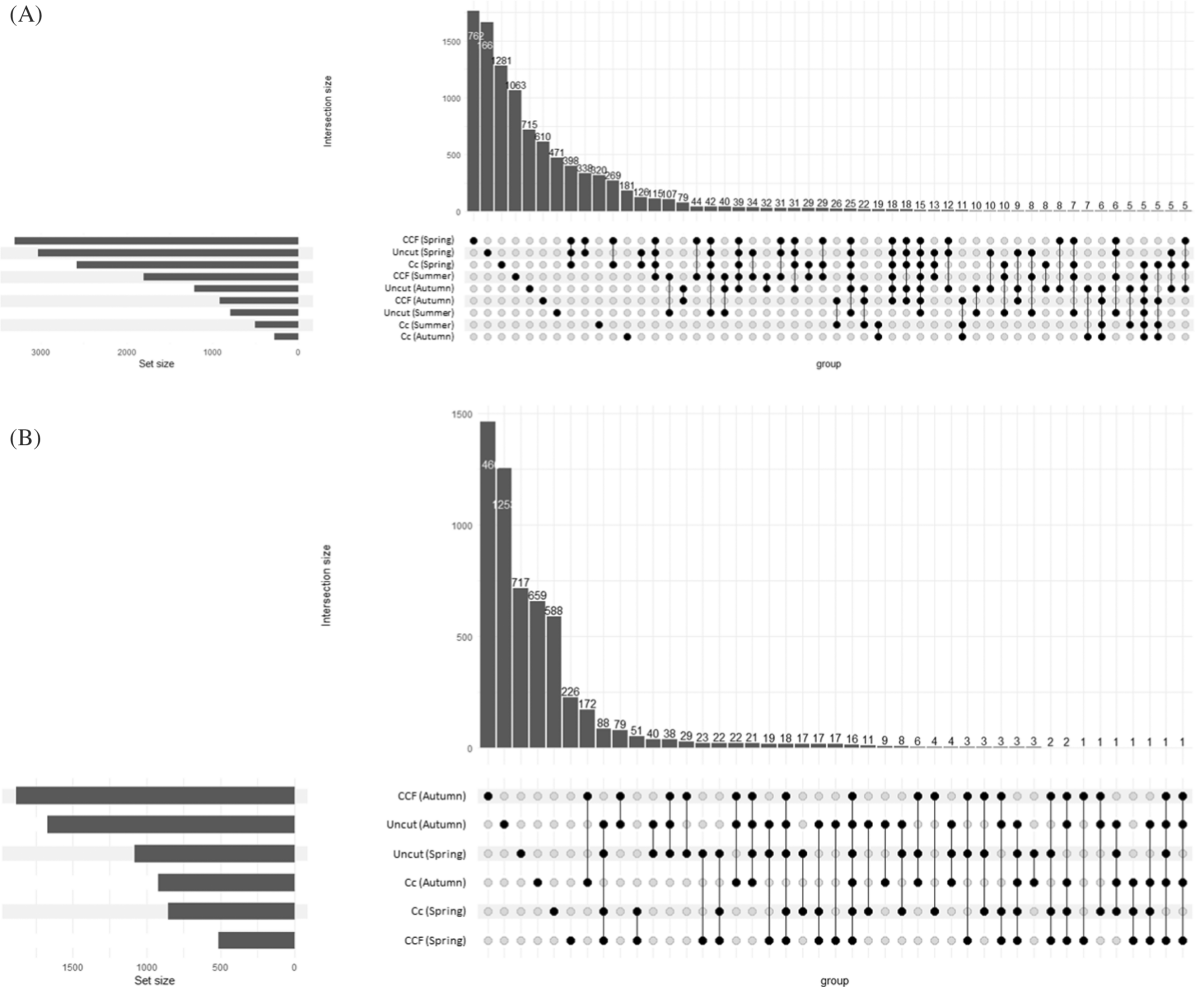
Upset plots for (A) bacterial and (B) fungal samples combined with season and harvesting intensity. The heights of the vertical bars at the top correspond to intersection size (connected dots), which is the number of significant ASV's shared among the corresponding samples. Horizontal bars indicate the set size. Cc, clear‐cut; CCF, continuous cover forestry.

The most common bacterial and fungal genera, according to relative abundance in different samples, are presented in Figure 4. The share of unassigned genera was high, especially in fungal samples in autumn. Bacterial genera *Pseudomonas* (Pseudomonadota) varied significantly with soil temperature (*p* <0.05). Additionally, genus *Roseiarcus* (Pseudomonadota) varied significantly with the season (*p* <0.05), and uncultured eubacterium WD260 varied significantly with WT. When examining the hierarchical clustering of the samples from the genus‐level heatmaps (Figure 5), bacterial and fungal samples cluster according to season and harvest intensity. Spring samples, in particular, cluster together, whereas there is slightly more variation in clustering with summer and autumn samples in both bacterial and fungal relative abundance. Examining the Upset plot (Figure 6A) revealed that in bacteria spring samples share the greatest number of ASVs. Despite the season and harvest intensity, the summer and autumn samples share much less ASVs. The least shared ASVs are with samples taken from clear‐cut in summer and autumn. In fungi (Figure 6B), the number of shared ASV's is more moderate. However, autumn samples (despite the harvest intensity) share the greatest number of ASVs. Spring and autumn samples taken from uncut, clear‐cut samples in spring and CCF samples in spring also all share a high amount of ASVs.

## DISCUSSION

We examined seasonal changes, the effects of prolonged drought periods during summer, and harvesting intensity on bacterial and fungal communities in drained forested peatlands over one growing season. We hypothesized seasonal changes in microbial communities. While the bacterial and fungal species richness and diversity did vary seasonally, there were only subtle changes in relative abundances in bacterial communities. It has been found that the diversity and community structure can be significantly different at the beginning of the growing season compared to the end (Santalahti et al., 2016; Shigyo et al., 2019; Wan et al., 2024). Additionally, in Wang et al. (2021) eukaryotic microbiomes, including fungi, showed significant shifts in community structure in rewetted peatlands, whereas in prokaryotic microbiomes were less prone to change. Although no seasonal variation was detected in the fungal relative abundances in our study, the drought period during the summer likely affected the fungal samples. This leads to an open question of whether the fungal communities are more vulnerable to drought.

During the summer drought, WT dropped 50 cm in clear‐cut, 48 cm in CCF and 44 cm in uncut forest. After the precipitation increased, WT rose by 30 cm in clear‐cut, 21 cm in CCF and 40 cm in uncut forest. Seasonal droughts have already been shown to decrease forest growth in boreal areas (Aakala & Kuuluvainen, 2011; Brecka et al., 2018; Keiluweit et al., 2016), but their effects on microbes and soil C processes are less studied (Potter et al., 2017). In our study, the drought potentially affected fungi and prevented high‐quality RNA for fungal sequencing in all experimental plots in the summer and autumn. Fungi have been reported to be more vulnerable to drought than bacteria (Allison & Treseder, 2008; Jaatinen et al., 2007; Krivtsov et al., 2006; Xue et al., 2021). Thus, increased drought may negatively affect fungi‐driven organic matter decomposition and tree growth. Furthermore, microbes have been shown to react to drought leading to changes in the soil C cycle and decomposition in other ecosystems and soil types such as grasslands (Metze et al., 2023) and silt‐clay loam soil (Xie et al., 2021). Our results might potentially indicate that drought may alter significantly microbially mediated C and nutrient cycling because fungi are the primary decomposers in boreal forest soils. However, the question of whether fungal functions and community structure are affected by drought remains open. It has been studied by Wang, Wang, et al. (2022) that in rewetted peatlands fungal and bacterial communities change significantly during wet and dry periods, indicating that microbial communities are susceptible to extreme weather conditions. Our samples included a high amount of unassigned fungal ASV's especially in autumn, which makes drawing further conclusions difficult. We argue that the matter should be further investigated and studied in the future on forested drained peatlands, as the drought evidently affected the sampling and further analysis with missing summer samples for fungi.

We found clear seasonal changes in microbial richness and diversity, but no significant differences between summer and autumn. The diversity and richness values were highest in spring for bacteria and autumn for fungi. Spring samples also showed more similarity for bacteria, whereas bacterial samples taken in summer and autumn were not as tightly related to each other. Fungal samples showed more systematic separation into spring and autumn. Seasonal changes in microbial diversity, richness and community composition have been examined and discovered in various environments and combined with other environmental factors like soil chemical properties and habitat characteristics can affect the microbial community richness and diversity (Luo et al., 2019; Shen et al., 2021; Solanki et al., 2024; Wan et al., 2024; Yu et al., 2024). Our results indicated that in drained peatland forests, both bacterial and fungal species richness and diversity are affected by seasonal changes, while bacterial diversity and richness are higher in spring, fungal richness and diversity are higher in autumn.

The common phyla found among fungi and bacteria are abundant in both peatland forests and peatlands overall (Generó, 2017; Kalam et al., 2020; Santalahti et al., 2016). Although Basidiomycota can be found especially in the upper layers of the peat (Lusa & Bomberg, 2021), they do not usually dominate the fungal community in peatlands (Thromann, 2006), whereas Basidiomycota are important and commonly found ectomycorrhizal fungi in boreal forests (Santalahti et al., 2016). In our study, the CCF and control communities were dominated by Basidiomycota in spring. However, the relative abundance of both Basidiomycota and Ascomycota declined on our sites in autumn. This might be due to the poorer quality of the autumn samples, leading to a lower portion of assigned ASV's in the autumn samples. Additionally, we did not find a statistically significant association between seasons, harvest intensity or any other environmental factor studied like WT. In Peltoniemi et al. (2009), Basidiomycota responded to the WT drawdown in peat soil and the Ascomycota became the dominant phyla after the treatment. Most soil fungi belong to Ascomycota and Basidiomycota, which form mutualistic relationships with plants and decompose recalcitrant organic C, including cellulose and polyphenolic compounds (Lynd et al., 2002).

In bacteria Proteobacteria, Actinobacteriota and Acidobacteriota are all common phyla in boreal peatlands and soils (Aislabie & Deslippe, 2013; Generó, 2017; Kolton et al., 2022; Lewin et al., 2016; Sun et al., 2014; Zhang et al., 2014). They can be acidophilic or aciduric bacteria (Curtis et al., 2002; Kalam et al., 2020), and they respond differently to WT drawdown (Kitson & Bell, 2020). Actinobacteriota have been noticed to potentially respond negatively to WT drawdown in wet and nutrient‐rich sites, but benefit in nutrient‐poor sites (Jaatinen et al., 2007). Proteobacteria can respond positively or negatively to drought (Potter et al., 2017), but the abundance increases during rewetting (He et al., 2015). It has been noticed that Acidobacteriota benefits from drainage in peat soil as they become the dominant group (Urbanová & Bárta, 2016). In our samples, the relative abundance of Acidobacteriota was quite stable but shifted in summer and autumn, becoming more abundant in CCF (summer) and uncut (autumn). Furthermore, the relative abundance of Proteobacteria increased across the harvest intensities, whereas the relative abundance of Actinobacteriota stayed quite stable or increased slightly towards summer and autumn. Additionally, we found that the relative abundance of phylum Myxococcota varied significantly between harvest intensity. Forest harvesting affects stand and ground vegetation (Kim et al., 2021), microclimate (Chroňáková et al., 2019), and WT which is controlled by interception and evapotranspiration (Sarkkola et al., 2013). The changes in the vegetation affect microbes through the soil–plant‐microbial interactions (Mundra et al., 2022; Schulp et al., 2008; Tedersoo et al., 2015; Wardle et al., 2004), and the removal of trees alters the light, temperature and moisture conditions. Mycococcota are unusual bacteria, capable of predation and fruiting body formation (Thiery & Kaimer, 2020; Wielgoss et al., 2019) and potentially photosynthesis (Li et al., 2023). Myxococcota can be found in various aerobic environments and their unique ecology allows them to exist in several types of environments (Reichenbach, 1999), which could explain our observations.

The relative abundance of bacteria was quite stable across the seasons and harvest intensities. The Acidobacteriae Subgroup_2, related to phosphorus ‘mining’ in nutrient‐poor soils (Jones et al., 2009; Mason et al., 2021), was abundant in spring, potentially indicating a higher springtime soluble phosphorus demand of plants. The genera related to the decomposition of recalcitrant C, such as cellulose (*Acidothermus*; Talia et al., 2012) and aromatic compounds (*Roseiarcus*; Man et al., 2022), were more abundant in spring than in autumn in the CCF and the uncut forest potentially reflecting the changes in the pool of labile substrates (Kirschbaum, 2013). Additionally, *Roseiarcus* showed significant differences in relative abundance when compared to season. The observations reflect the soil acidity and the ground vegetation of the management plots since most of these genera are concerned as acidophilic (e.g., *Acinetobacter*, *Chloroflexi*, AD3) and often regarded as members of mosses' or shrubs' microbiome associated with N cycling in low N environments (e.g., *Candidatus* S*olibacter*, candidate phylum WPS‐2) (Holland‐Moritz et al., 2018; Huber et al., 2017; Jenkins et al., 2009; Köhler et al., 2021; Kolton et al., 2022; Rodriguez‐Mena et al., 2022; Tian et al., 2019). The relative abundance of genus *Pseudomonas* was significantly different when compared with soil temperature, and the relative abundance of a genus of uncultured eubacterium WD260 differed significantly when compared with WT. Fungal genera did not differ significantly when compared with environmental factors. However, the relative abundance of ectomycorrhizal genus *Asterostroma* was high in all harvest intensities in spring but declined in the autumn.

To conclude, this study investigated the microbial communities and drought in the topsoil layer of drained forested boreal peatlands. Despite the limited research on this topic, there is a growing demand for information due to new forestry practices and the impacts of climate change. We found some strong indications of seasonal changes in bacterial and fungal community diversity and species richness. Furthermore, some differences were observed in bacterial phyla and genera based on harvesting intensity, soil temperature, season and WT. We found some potential indications of the effects of drought, but due to the low quality of fungal samples in summer and autumn we could not draw further conclusions about whether the fungal abundance was affected by the drought. Additionally, there were no indications that the drought affected the bacterial relative abundance. Since our study was carried out only over one growing season, we suggest that similar longer‐term studies in varying weather conditions should be conducted. As the seasonal droughts are predicted to increase in boreal areas due to climate change‐promoted increase in temperature and evapotranspiration, as well as due to more irregular precipitation patterns (Diffenbaugh & Field, 2013; Donat et al., 2016; Gauthier et al., 2015; Ge et al., 2010; Reyer et al., 2015), further examination of the effects of prolonged drought periods on the microbial communities is essential.

## AUTHOR CONTRIBUTIONS

**Oona Hillgén:** Investigation; conceptualization; writing – review and editing; visualization; formal analysis; data curation; writing – original draft. **Marjo Palviainen:** Writing – review and editing; writing – original draft; validation; supervision. **Annamari Laurén:** Writing – review and editing; writing – original draft; validation; supervision. **Mari Könönen:** Investigation; conceptualization; writing – review and editing; formal analysis; validation. **Anne Ojala:** Conceptualization; writing – review and editing; funding acquisition; methodology; validation; supervision. **Jukka Pumpanen:** Conceptualization; writing – review and editing; funding acquisition; project administration; methodology; validation; supervision. **Elina Peltomaa:** Investigation; conceptualization; funding acquisition; writing – original draft; writing – review and editing; visualization; project administration; formal analysis; methodology; validation; supervision; data curation.

## CONFLICT OF INTEREST STATEMENT

The authors declare no conflict of interest.

## Supporting information


**Figure S1.** Daily precipitation (blue; left axis) and air temperature (red; right axis) during the growing season of 2021. The sampling times for soil microbial community analysis are marked with black arrows.

**Figure S2.** The Y1‐axis titled ‘Tags Number’ means the number of tags; ‘Total tags’ (red bars) is the number of effective tags; ‘Taxon Tags’ (blue bars) is the number of annotated tags; ‘Unclassified Tags’ (green bars) is the number of unannotated tags; ‘Unique Tags’ (orange bars) is the number of tags with a frequency of 1 and only occurs in one sample. The Y2‐axis titled ‘OTUs Numbers’ means the number of OTUs, which are displayed as ‘OTUs’ (purple bars) to identify the numbers of OTUs in different samples. Panel A is for bacterial (16S) samples and panel B for fungal (ITS2) samples.

**Table S1.** The amplicon was sequenced on Illumina paired‐end platform to generate 250‐bp paired‐end raw reads (raw PE) and then merged and pretreated to obtain Clean Tags. The chimeric sequences in Clean Tags were detected and removed to obtain the Effective Tags, which can be used for subsequent analysis. The summarizations obtained in each step of data processing are shown in the table.

**Table S2.** Good's coverage and alpha diversity and richness indices per sample for (A) bacteria and (B) fungi. CC, clear cut; CCF, continuous cover forestry. A, B and C mark the sample replicants.

## Data Availability

The data that support the findings of this study are openly available in Sequence Read Archive (SRA) at BioProject PRJNA1095915.
