# Supplementary material for: Subtle changes in topsoil microbial communities of drained forested peatlands after prolonged drought
Source: Environ Microbiol Rep. 2024 Nov 7;16(6):e70041. doi: 10.1111/1758-2229.70041 (PMC11544035; doi:10.1111/1758-2229.70041)
Supplement: Supplementary file 1 — Figure S1. Daily precipitation (blue; left axis) and air temperature (red; right axis) during the growing season of 2021. The sampling times for soil microbial community analysis are marked with black arrows. [file EMI4-16-e70041-s001.pdf]

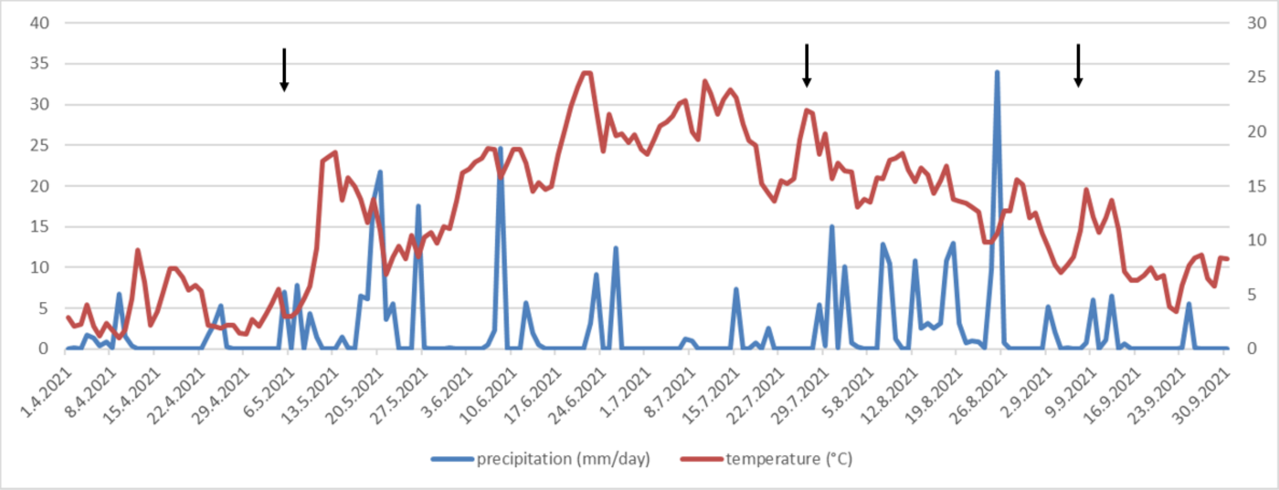

Supplemental Figure 1. Daily precipitation (blue; left axis) and air temperature (red; right axis) during the growing season of 2021. The sampling times for soil microbial community analysis are marked with black arrows.
