# Supplementary material for: Subtle changes in topsoil microbial communities of drained forested peatlands after prolonged drought
Source: Environ Microbiol Rep. 2024 Nov 7;16(6):e70041. doi: 10.1111/1758-2229.70041 (PMC11544035; doi:10.1111/1758-2229.70041)
Supplement: Supplementary file 2 — Figure S2. The Y1‐axis titled ‘Tags Number’ means the number of tags; ‘Total tags’ (red bars) is the number of effective tags; ‘Taxon Tags’ (blue bars) is the number of annotated tags; ‘Unclassified Tags’ (green bars) is the number of unannotated tags; ‘Unique Tags’ (orange bars) is the number of tags with a frequency of 1 and only occurs in one sample. The Y2‐axis titled ‘OTUs Numbers’ means the number of OTUs, which are displayed as ‘OTUs’ (purple bars) to identify the numbers of OTUs in different samples. Panel A is for bacterial (16S) samples and panel B for fungal (ITS2) samples. [file EMI4-16-e70041-s004.pdf]

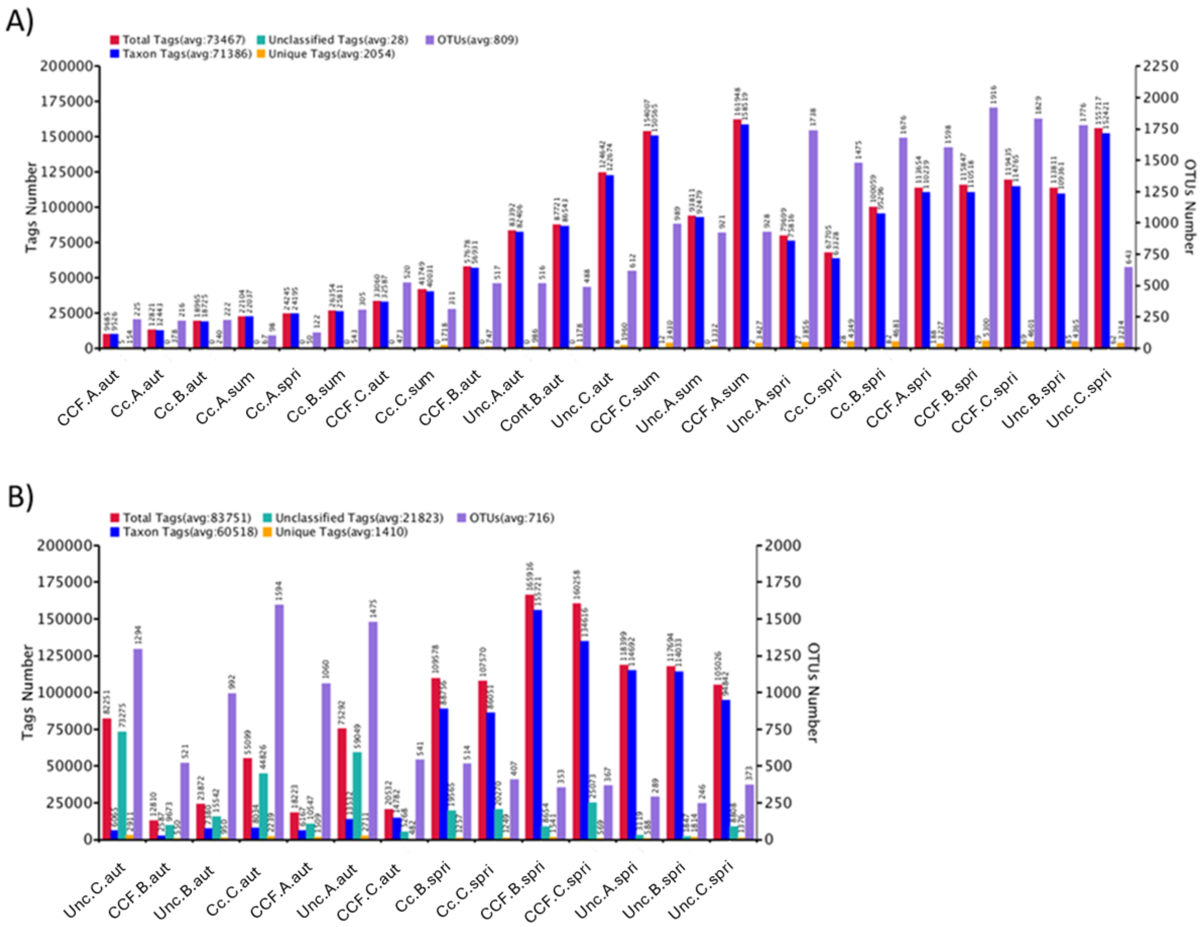

Supplemental Figure 2. The Y1-axis titled "Tags Number" means the number of tags; Total tags" (red bars) is the number of effective tags; Taxon Tags" (blue bars) is the number of annotated tags; Unclassified Tags" (green bars) is the number of unannotated tags; Unique Tags" (orange bars) is the number of tags with a frequency of 1 and only occurs in one sample. The Y2-axis titled "OTUs Numbers" means the number of OTUs, which are displayed as "OTUs" (purple bars) to identify the numbers of OTUs in different samples. Panel A is for bacterial (16S) samples and panel B for fungal (ITS2) samples.
