# Supplementary material for: Subtle changes in topsoil microbial communities of drained forested peatlands after prolonged drought
Source: Environ Microbiol Rep. 2024 Nov 7;16(6):e70041. doi: 10.1111/1758-2229.70041 (PMC11544035; doi:10.1111/1758-2229.70041)
Supplement: Supplementary file 3 — Table S1. The amplicon was sequenced on Illumina paired‐end platform to generate 250‐bp paired‐end raw reads (raw PE) and then merged and pretreated to obtain Clean Tags. The chimeric sequences in Clean Tags were detected and removed to obtain the Effective Tags, which can be used for subsequent analysis. The summarizations obtained in each step of data processing are shown in the table. [file EMI4-16-e70041-s003.pdf]

Supplemental Table 1. The amplicon was sequenced on Illumina paired-end platform to generate 250 bp paired-end raw reads (Raw PE), and then merged and pretreated to obtain Clean Tags. The chimeric sequences in Clean Tags were detected and removed to obtain the Effective Tags which can be used for subsequent analysis. The summarizations obtained in each step of data processing are shown in the table.

Sequence	Sample Name	Raw PE(#)	Combined(#	Qualified(#	Nochime(#	Base(nt)	AvgLen(nt	Q20	Q30	GC%	Effective%
**Bacteria**	Clear-cut.A.spring	25,613	24,790	24,553	24,245	10,212,587	421	98,72	95,5	54,44	94,66
**16S**	Clear-cut.B.spring	142,367	123,865	122,560	100,059	41,088,338	411	98,26	94,5	56,4	70,28
	Clear-cut.C.spring	112,668	98,343	97,265	67,705	27,774,262	410	98,26	94,49	56,67	60,09
	Clear-cut.A.summe	23,638	22,656	22,391	22,104	9,411,225	426	98,61	95,24	54,47	93,51
	Clear-cut.B.summe	29,807	28,838	28,523	26,354	11,119,209	422	98,68	95,48	54,7	88,42
	Clear-cut.C.summe	51,798	49,625	48,884	41,749	17,536,769	420	98,56	95,07	51,91	80,6
	Clear-cut.A.atumn	16,178	15,560	15,420	12,821	5,355,691	418	98,71	95,49	51,46	79,25
	Clear-cut.B.atumn	20,697	20,054	19,807	18,965	8,109,053	428	98,67	95,35	53,72	91,63
	CCF.A.spring	146,785	127,062	125,720	113,654	46,898,652	413	98,2	94,28	56,28	77,43
	CCF.B.spring	154,583	134,401	132,881	115,847	47,590,185	411	98,29	94,53	56,57	74,94
	CCF.C.spring	155,072	134,545	132,741	119,435	49,270,261	413	98,31	94,51	56,3	77,02
	CCF.A.summer	183,701	175,920	174,217	161,948	67,185,527	415	98,62	95,21	56,71	88,16
	CCF.C.summer	186,427	178,218	176,375	154,007	63,914,569	415	98,57	95,15	56,46	82,61
	CCF.A.atumn	11,265	10,602	10,459	9,685	4,097,502	423	98,74	95,51	52,99	85,97
	CCF.B.atumn	75,398	71,248	70,420	57,678	24,258,485	421	98,6	95,35	55,75	76,5
	CCF.C.atumn	37,956	36,530	36,164	33,060	13,825,815	418	98,74	95,6	54,62	87,1
	Uncut.A.spring	111,152	96,676	95,610	79,699	32,775,531	411	98,22	94,35	56,34	71,7
	Uncut.B.spring	147,906	127,263	125,701	113,811	46,710,906	410	98,19	94,29	56,26	76,95
	Uncut.C.spring	193,076	165,464	163,719	155,717	63,851,610	410	98,27	94,49	56,47	80,65
	Uncut.A.summer	108,449	104,769	103,880	93,811	40,032,883	427	98,62	95,19	54,97	86,5
	Uncut.A.atumn	91,711	88,732	87,626	83,392	35,680,923	428	98,69	95,43	54,61	90,93
	Uncut.B.atumn	101,816	97,780	97,022	87,721	36,244,688	413	98,72	95,62	57,16	86,16
	Uncut.C.atumn	146,493	140,234	138,855	124,642	51,942,510	417	98,56	95,06	56,55	85,08
	Uncut.A.summer	108,449	104,769	103,880	93,811	40,032,883	427	98,62	95,19	54,97	86,5
	Uncut.C.atumn	146,493	140,234	138,855	124,642	51,942,510	417	98,56	95,06	56,55	85,08
**Fungi**	Clear-cut.B.spring	124,329	119,337	117,088	109,578	37,913,063	346	98,94	96,35	48,68	88,14
**ITS2**	Clear-cut.C.spring	118,705	114,734	114,237	107,570	36,520,937	340	98,96	96,49	49,94	90,62
	Clear-cut.C.atumn	117,652	58,011	56,222	55,099	7,831,878	142	99,15	97,33	48,71	46,83
	CCF.B.spring	199,877	192,810	176,896	165,916	58,215,937	351	98,99	96,44	45,64	83,01
	CCF.C.spring	183,816	175,758	173,973	160,258	53,456,678	334	99,04	96,76	46,08	87,18
	CCF.A.atumn	98,594	62,886	19,370	18,223	5,286,067	290	99,09	96,96	50,77	18,48
	CCF.B.atumn	27,296	18,995	13,309	12,810	4,719,639	368	98,64	95,48	46,09	46,93
	CCF.C.atumn	108,271	59,199	23,008	20,532	6,896,484	336	99,17	96,98	47,1	18,96
	Uncut.A.spring	184,672	179,486	129,546	118,399	41,680,519	352	98,93	96,26	46,48	64,11
	Uncut.B.spring	127,196	123,669	122,520	117,694	41,054,021	349	98,94	96,32	47,15	92,53
	Uncut.C.spring	117,565	113,865	110,784	105,026	35,314,282	336	99,02	96,66	48,12	89,33
	Uncut.A.atumn	181,818	79,785	77,105	75,292	10,028,200	133	98,79	96,72	46,61	41,41
	Uncut.B.atumn	56,408	35,925	24,906	23,872	5,392,443	226	99,29	97,37	48,81	42,32
	Uncut.C.atumn	192,254	86,839	83,069	82,251	8,353,768	102	98,57	96,53	48,2	42,78
