# Supplementary material for: Subtle changes in topsoil microbial communities of drained forested peatlands after prolonged drought
Source: Environ Microbiol Rep. 2024 Nov 7;16(6):e70041. doi: 10.1111/1758-2229.70041 (PMC11544035; doi:10.1111/1758-2229.70041)
Supplement: Supplementary file 4 — Table S2. Good's coverage and alpha diversity and richness indices per sample for (A) bacteria and (B) fungi. CC, clear cut; CCF, continuous cover forestry. A, B and C mark the sample replicants. [file EMI4-16-e70041-s002.pdf]

Supplemental table 2: Good's coverage and Alpha diversity and richness indices per sample for A) bacteria and B) fungi. Cc refers to clear cut and CCF to continuous cover forestry. A, B and C are marking the sample replicants.

A)

	Observed species	Chao1	se.chao1	ACE	se.ACE	Shannon	Simpson	Goods Coverage
**Cc.A.spring**	65	74.23077	6.164647	76.44200	4.117190	2.415824	0.8479068	0.997979 0
**Cc.B.spring**	1193	1462.26271	37.280122	1492.26724	18.379257	5.885424	0.9844874	0.9549072
**Cc.C.spring**	1082	1316.00476	34.544194	1349.76892	17.716031	5.814416	0.986944	0.9603385
**CCF.A.spring**	764	972.13333	36.648989	996.83103	16.171973	5.639940	0.9918855	0.9717065
**CCF.B.spring**	1300	1650.10728	44.341714	1698.72104	20.650924	6.165668	0.9940433	0.9459391
**CCF.C.spring**	979	1242.55346	40.731456	1247.39149	17.574919	5.961312	0.9936123	0.9633700
**Uncut.A.spring**	1268	1536.89362	37.282029	1557.08195	18.541595	6.338014	0.9961407	0.9550335
**Uncut.B.spring**	872	1161.10791	45.469629	1180.94618	18.220937	5.814727	0.994477	0.9641278
**Uncut.C.spring**	725	825.30928	22.192879	817.72977	13.605182	5.759971	0.9937175	0.9823165
**Cc.A.summer**	72	84.21429	7.433585	89.43905	4.533185	2.837238	0.9032591	0.9976001
**Cc.B.summer**	202	261.00000	20.370228	269.25398	9.002734	3.737141	0.9618566	0.9925477
**Cc.C.summer**	186	267.05882	30.824453	260.12989	8.685682	3.889431	0.960197	0.9933055
**CCF.A.summer**	636	875.40000	46.971498	835.35577	14.868940	5.447440	0.9917381	0.9760010
**CCF.C.summer**	621	734.47826	24.690678	744.29876	13.532174	5.530565	0.9924349	0.9816850
**Uncut.A.summer**	409	587.41892	37.266334	597.08139	13.138658	3.336946	0.8790638	0.9794114
**Cc.A.autumn**	144	153.37500	5.165970	160.89634	6.090261	3.231195	0.9350569	0.9968422
**Cc.B.autumn**	145	179.87097	13.362680	184.59106	7.202277	2.420114	0.7992015	0.9940634
**CCF.A.autumn**	159	161.05263	1.888639	164.68473	5.577085	2.972742	0.8941399	0.9983580
**CCF.B.autumn**	273	415.80000	35.041297	447.81933	13.307960	3.224263	0.9258259	0.9848427
**CCF.C.autumn**	366	469.84146	23.780671	491.34495	11.019730	3.378874	0.9314697	0.9834533
**Uncut.A.autumn**	244	390.87234	36.517472	442.79740	13.053240	3.020396	0.8949503	0.9850954
**Uncut.B.autumn**	304	415.72414	33.931953	413.05124	10.408476	4.731478	0.9835380	0.9897689
**Uncut.C.autumn**	259	341.65000	29.773119	322.72778	9.204503	4.515947	0.9811892	0.9926740

B)

	Observed species	Chao1	se.chao1	ACE	se.ACE	Shannon	Simpson	Goods Coverage
**Cc.B.spring**	507	609.2381	25.2095317	593.6631	11.826733	3.867201	0.9396275	0.9939663
**Cc.C.spring**	328	367.7826	13.3772749	372.2880	9.441306	3.667688	0.9416210	0.9967715
**CCF.B.spring**	252	313.0000	20.7111752	316.1061	9.092963	3.496473	0.9320355	0.9967715
**CCF.C.spring**	244	315.0909	22.7348113	314.9220	9.072855	3.756720	0.9493917	0.9944956
**Uncut.A.spring**	306	330.8485	10.2342779	330.3745	8.970323	3.692062	0.9398658	0.9978300
**Uncut.B.spring**	142	194.9286	23.0984446	206.2601	7.992507	3.384734	0.9391693	0.9979359
**Uncut.C.spring**	292	315.6500	9.4507267	329.8774	9.142894	4.137934	0.9643029	0.9976712
**Cc.C.autumn**	823	909.5789	19.1291765	911.5804	14.431972	5.153214	0.9822990	0.9925373
**CCF.A.autumn**	852	957.6250	21.2395545	975.0736	15.050386	5.124353	0.9831163	0.9910024
**CCF.B.autumn**	425	425.3544	0.6542913	427.8747	9.926210	4.009554	0.9404299	0.9995766

**CCF.C.autumn**	607	674.7975	17.4284238	677.4708	12.739683	4.871140	0.9820701	0.9963480
**Uncut.A.autumn**	735	819.8391	20.1136786	815.4582	14.032265	4.939325	0.9765202	0.9935429
**Uncut.B.autumn**	539	594.9429	15.5649785	594.5300	11.773386	4.551405	0.9684034	0.9952895
**Uncut.C.autumn**	540	618.9062	20.5844094	615.3595	12.283734	4.697213	0.9771289	0.9946544
